# Performance Testing of a Magnetically Suspended Double Gimbal Control Moment Gyro Based on the Single Axis Air Bearing Table

**DOI:** 10.3390/s120709129

**Published:** 2012-07-03

**Authors:** Peiling Cui, Huijuan Zhang, Ning Yan, Jiancheng Fang

**Affiliations:** 1 School of Instrumentation Science and Optoelectronics Engineering, Beijing University of Aeronautics and Astronautics, Beijing 100191, China; E-Mails: zhanghjqy@126.com (H.Z.); n_yann@sina.com (N.Y.); fangjiancheng@buaa.edu.cn (J.F.); 2 Science and Technology on Inertial Laboratory, Beijing 100191, China; 3 Fundamental Science on Novel Inertial Instrument & Navigation System Technology Laboratory, Beijing 100191, China

**Keywords:** magnetically suspended double gimbal control moment gyroscope (MSDGCMG), attitude control, single axis air bearing table, experiment

## Abstract

Integrating the advantage of magnetic bearings with a double gimble control moment gyroscope (DGCMG), a magnetically suspended DGCMG (MSDGCMG) is an ideal actuator in high-precision, long life, and rapid maneuver attitude control systems. The work presented here mainly focuses on performance testing of a MSDGCMG independently developed by Beihang University, based on the single axis air bearing table. In this paper, taking into sufficient consideration to the moving-gimbal effects and the response bandwidth limit of the gimbal, a special MSDGCMG steering law is proposed subject to the limits of gimbal angle rate and angle acceleration. Finally, multiple experiments are carried out, with different MSDGCMG angular momenta as well as different desired attitude angles. The experimental results indicate that the MSDGCMG has a good gimbal angle rate and output torque tracking capabilities, and that the attitude stability with MSDGCMG as actuator is superior to 10^−3^°/s. The MSDGCMG performance testing in this paper, carried out under moving-base condition, will offer a technique base for the future research and application of MSDGCMGs.

## 1. Introduction

Because a control moment gyroscope (CMG) is capable of generating large control torques and storing large angular momentum over long periods of time, it is often favored for precision pointing and tracking control of agile spacecraft in low Earth orbit and momentum management of large space vehicles [1,2]. Depending on the gimbal axes, a CMG is distinguished as either a single gimbal CMG (SGCMG) or double gimbal CMG (DGCMG) [3]. Compared with SGCMG, DGCMG has many advantages such as the output control torques with two degrees of freedom, nearly spherical angular momentum envelope, good configuration efficiency, no striking singularity problem, low steering law computation complexity, and so on. Meanwhile, the compatibility of the steering law is quite good when part of the DGCMGs fail [4,5].

The manner of support of the high-rate rotor is a deciding factor in the comprehensive performance of a CMG [6]. Considering the manner of support, CMGs are divided into mechanical CMGs and magnetically suspended CMGs (MSCMGs). In comparison with the mechanical CMGs, MSCMGs have the merits of being friction-free, long life, and so on. Meanwhile, the control accuracy is improved by active vibration control techniques. With the same angular momentum, the size and weight of a CMG is effectively reduced for the reason of a high-rate magnetically suspended rotor [7,8]. Incorporating the advantage of magnetic bearings with that of DGCMGs, magnetically suspended DGCMGs (MSDGCMG) will become an ideal solution to realize high precision, long life, and rapid maneuver attitude control for spacecraft.

In the 1960s, the US devoted itself to the research and development of DGCMGs. In 1973, three orthogonally mounted DGCMGs were firstly employed on NASA's Skylab as the main actuator [9–11], and four parallel mounted DGCMGs were applied on the International Space Station (ISS) in 1998 [12–14]. Up to the present, DGCMGs have been successfully employed for a variety of space missions. However, the related research in the open literature on attitude control systems with DGCMGs as attitude actuators was based on mechanical-bearing DGCMGs. In the aspect of the investigation of MSDGCMG, Beihang University has been devoted to developing the MSDGCMG since 1998, and has successfully developed the first MSDGCMG in the world. Up to the present, all the research on MSDGCMG is carried out on the resting base. They include: optimization design of a magnetic suspended gyroscope rotor [6], compensation of moving-gimbal effects [15], AMB vibration control for structural resonance [16], and so on. However, there still exist various practical as well as theoretical issues inherent in the performance of MSDGCMGs installed on the moving-base and the use of MSDGCMGs for attitude control.

In this paper, we focus our attention on the performance test of MSDGCMGs under the moving-base condition, such as gimbal angle rate and MSDGCMG output torque tracking capacity as well as attitude stability with an MSDGCMG as an attitude actuator. The remainder of this paper is organized as follows: Section 2 briefly introduces the mathematical model of the single axis air bearing table. In Section 3, a parameter segment control law is used for the rapid maneuver and quick stability subjected to the control torque saturation. In view of the moving-gimbal effect on the stability of the magnetic bearing and the response bandwidth limit of the MSDGCMG gimbal, Section 4 presents a special steering law subject to the constraints of MSDGCMG gimbal angle rate and acceleration. Section 5 gives a detailed description of the semi-physical simulation platform of the MSDGCMG performance testing experiments. In Section 6, experiments and the corresponding results analysis are given in detail. Finally, some conclusions are drawn in Section 7.

## 2. Mathematical Model of the Single Axis Air Bearing Table

The objective of this section is to give a mathematical model of the single axis air bearing table for developing the attitude control law in the next section. The models are introduced as follows, including the dynamic model and the error quaternion kinematics differential equation.

### 2.1. Dynamic Model of the Single Axis Air Bearing Table

In the experiment, the single axis air bearing table is utilized for simulating the rotational motion of a certain physical axis of the satellite, and according to the feature of large angle and rapid maneuver, the pitch axis (*Y*) is chosen as the physical axis. The dynamic model of the single axis air bearing table is governed by the following differential equation:

(1) $$J\dot{\omega }=u+T_{d}$$

where *ω* denotes the air bearing table rotation angle rate, *J* is the moment of inertia of the air bearing table, *u* is the control torque input, and *T_d_* is the disturbance torque.

### 2.2. Kinematics Equation of the Single Axis Air Bearing Table

Assuming that the “3-1-2” Euler angle rotation is adopted, the relationship between the attitude quaternion and Euler angle is described as follows:

(2) $$q=\left[\begin{array}{c} q_{0}\\ q_{1}\\ q_{2}\\ q_{3} \end{array}\right]=\left[\begin{array}{l} \cos\frac{\varphi }{2}\cos\frac{\theta }{2}\cos\frac{\psi }{2}-\sin\frac{\varphi }{2}\sin\frac{\theta }{2}\sin\frac{\psi }{2}\\ \sin\frac{\varphi }{2}\cos\frac{\theta }{2}\cos\frac{\psi }{2}-\cos\frac{\varphi }{2}\sin\frac{\theta }{2}\sin\frac{\psi }{2}\\ \cos\frac{\varphi }{2}\sin\frac{\theta }{2}\cos\frac{\psi }{2}+\sin\frac{\varphi }{2}\cos\frac{\theta }{2}\sin\frac{\psi }{2}\\ \sin\frac{\varphi }{2}\sin\frac{\theta }{2}\cos\frac{\psi }{2}+\cos\frac{\varphi }{2}\cos\frac{\theta }{2}\sin\frac{\psi }{2} \end{array}\right]$$

where *q*_0_ denotes the scalar part of the quaternion, *q̄* = [*q*_1_ *q*_2_ *q*_3_]*^T^* is the vector part of the quaternion. *φ, θ* and *ψ* denote the roll, pitch and yaw angle, respectively. Considering the case of *φ* = *0* and *ψ* = *0* for the single axis air bearing table, then the attitude of the air bearing table is rewritten in the form of quaternion as follows:

(3) $$q={\left[\begin{array}{cccc} \cos\frac{\theta }{2} & 0 & \sin\frac{\theta }{2} & 0 \end{array}\right]}^{T}$$

If the Euler angle *θ_c_* defines the command air bearing table rotation angle, the corresponding command attitude quaternion is given as:

(4) $$q_{c}={\left[\begin{array}{cc} q_{c0} & {\bar{q}}_{c}^{T} \end{array}\right]}^{T}={\left[\begin{array}{cccc} \cos\frac{\theta_{c}}{2} & 0 & \sin\frac{\theta_{c}}{2} & 0 \end{array}\right]}^{T}$$

Then the error quaternion, which represents the error between the current attitude quaternion and the command attitude quaternion, is defined as:

(5) $$q_{ev}=\left[\begin{array}{cc} q_{c0} & {\bar{q}}_{c}^{T}\\ -{\bar{q}}_{c} & -\left[{\bar{q}}_{c}\times \right]+q_{c0}I_{3\times 3} \end{array}\right]q$$

Substituting (3) and (4) into (5) gives the error quaternion:

(6) $$q_{ev}=\left[\begin{array}{c} q_{e0}\\ q_{e1}\\ q_{e2}\\ q_{e3} \end{array}\right]=\left[\begin{array}{c} \cos(\frac{\theta -\theta_{c}}{2})\\ 0\\ \sin(\frac{\theta -\theta_{c}}{2})\\ 0 \end{array}\right]$$

and then the error quaternion kinematics differential equation is expressed as:

(7) $${\dot{q}}_{ev}=\frac{1}{2}\left[\begin{array}{c} -q_{e2}\cdot \omega \\ 0\\ q_{e0}\cdot \omega \\ 0 \end{array}\right]=\left[\begin{array}{c} -\sin(\frac{\theta -\theta_{c}}{2})\cdot \omega \\ 0\\ \cos(\frac{\theta -\theta_{c}}{2})\cdot \omega \\ 0 \end{array}\right]$$

## 3. Parameter Segment Attitude Control Law

The attitude control system is nonlinear. Simultaneously, there exists strong coupling among the air bearing table, high-rate magnetically suspended rotor and the gimbal of the MSDGCMG. Moreover, the single axis air bearing table is subject to disturbance torques and the saturation limit of the control torque, and the moment of inertia of the air bearing table is uncertain for the reason of the rotation of the inner and outer gimbal. Considering the characteristics of the attitude control system mentioned above, a parameter segment control law is used in this paper and the parameters of attitude control law are chosen according to the error-angle to realize rapid maneuvers and quick stability.

In the light of the dynamics and kinematics Equations (1,7), the control torque of pitch axis of the air bearing table is derived by referring to [17]:

(8) $$\tau =-\frac{1}{2}q_{e2}-\left(k+\varepsilon \right)\left(\omega +aq_{e2}\right)-Y\hat{J}$$

where *k, a* and *ε* are positive scalars to be properly determined. The derivative of the moment of inertia estimation *Ĵ* is calculated as:

(9) $$\dot{\hat{J}}=gY\left(\omega +aq_{e2}\right)$$

where *g* is a positive scalar and:

(10) $$Y=a{\dot{q}}_{e2}=\frac{1}{2}aq_{e0}\omega$$

For the purpose of rapid maneuvering and quick stability, the parameters *k* and *a* are chosen in accordance with the error-angle *θ_e_, θ_e_* = *θ* − *θ_c_*. When *θ_e_* is large, particular attention is paid to improving the response speed, whereas when *θ_e_* is small, the main emphasis is placed on constraining the attitude overshoot, reducing the settling time as well as guaranteeing the stability of the system. Therefore, *k* and *a* are selected according to:

(11) $$k=\left\{ \begin{array}{l} \begin{array}{cc} k_{1} & \begin{array}{cc} if & \theta_{e1}\leq \left|\theta_{e}\right| \end{array} \end{array}\\ \begin{array}{cc} k_{2} & \begin{array}{cc} if & \theta_{e2}<\left|\theta_{e}\right|<\theta_{e1} \end{array} \end{array}\\ \begin{array}{cc} k_{3} & \begin{array}{cc} if & \left|\theta_{e}\right|\leq \theta_{e2} \end{array} \end{array} \end{array}\right.$$

(12) $$a=\left\{ \begin{array}{l} \begin{array}{cc} a_{1} & \begin{array}{cc} if & \theta_{e1}\leq \left|\theta_{e}\right| \end{array} \end{array}\\ \begin{array}{cc} a_{2} & \begin{array}{cc} if & \theta_{e2}<\left|\theta_{e}\right|<\theta_{e1} \end{array} \end{array}\\ \begin{array}{cc} a_{3} & \begin{array}{cc} if & \left|\theta_{e}\right|\leq \theta_{e2} \end{array} \end{array} \end{array}\right.$$

Where *θ_e1_* and *θ_e2_* are the piecewise points of the error-angle.

Taking account of the torque output capacity of the MSDGCMG, the control torque *τ* becomes constrained with the saturation limit *U*_max_, then the command control torque is:

(13) $$u=\{\begin{array}{cc} \tau & \begin{array}{cc} if & \left|\tau \right|<U_{\max} \end{array}\\ \tau \frac{U_{\max}}{\left|\tau \right|} & \begin{array}{cc} if & \left|\tau \right|\geq U_{\max} \end{array} \end{array}$$

Due to the coupling between the air bearing table, MSDGCMG gimbal and high-rate rotor, the movement of the disturbed rotor becomes more complex, which aggravates the runout of the rotor and even endangers the system stability. What's more, the quicker the angle velocity of the air bearing table is, the more serious the influence on the high-rate magnetically suspended rotor becomes. The maximum angle velocity of the air bearing table is closely related to *U*_max_. Therefore, a smaller value than the maximum output torque of MSDGCMG is used as the saturation limit *U*_max_ to ensure MSDGCMG steadiness in our experiments.

## 4. MSDGCMG Steering Law

The command control torque is derived by means of the above mentioned attitude control algorithm, and then a steering law is presented in this section to calculate the command gimbal angle rate on the basis of the command control torque and the current gimbal angle. The chief points of this section include: the introduction of the MSDGCMG and the corresponding coordinate system, MSDGCMG torque equation and the detailed development of the steering law.

### 4.1. MSDGCMG and the Corresponding Coordinate System

The MSDGCMG consists of inner and outer gimbal servo systems as well as a high-rate rotor system. The high-rate rotor is mounted in the stator housing and provides a constant angular momentum. With a specific rotation of inner and outer gimbal, the vector direction of the rotor angular momentum is changed to produce the desired gyroscope torques to meet the requirements of the attitude control. The main parameters of the MSDGCMG used in this paper are:

1. Angular momentum: ≥15 Nms.
2. Maximum output torque: ≥10 Nm.
3. Nominal rotor speed: 30,000 r/min.
4. Gimbal angle range: 0°∼360°.

**Figure 1.**
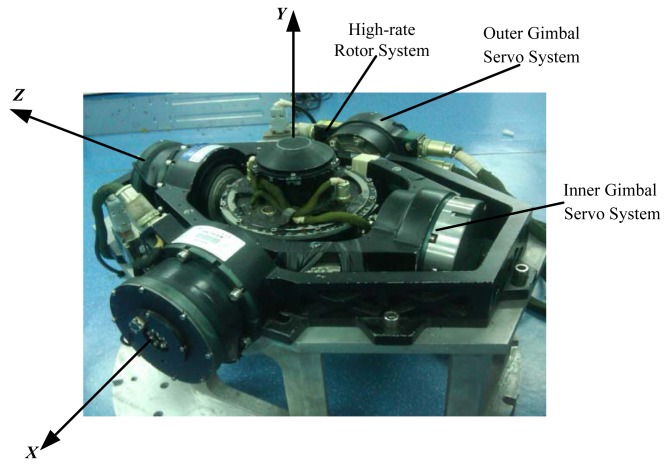
Schematic Diagram of the MSDGCMG Coordinate System.

Furthermore, the circuit box of MSDGCMG is used to control the inner and outer gimbal servo systems as well as the high-rate rotor system. Assume the inner gimbal axis, outer gimbal axis and rotor axis are perpendicular to each other at the initial time, then the MSDGCMG coordinate system is defined as in Figure 1, outer gimbal axis as *X* axis, inner gimbal axis as *Z* axis, and *Y* axis in agreement with the Right-handed Rule. When the rotor angular momentum revolves about the positive half of the *Z* axis, the inner gimbal angle changes *α*. When the rotor angular momentum revolves about the positive half of the *X* axis, the outer gimbal angle changes by −*β*.

### 4.2. MSDGCMG Torque Equation

In this paper, MSDGCMG is put on the single axis air bearing table. At the initial time, the *Y* axis of the MSDGCMG coordinate system is parallel to the rotation (pitch) axis of the air bearing table, whereas the inner gimbal axis (*Z* axis) and the outer gimbal axis (*X* axis) are perpendicular to the pitch axis. Consequently, the installation matrix of MSDGCMG is a 3 × 3 identity matrix. According to the definition of the MSDGCMG coordinate system and the installation matrix, the CMG angular momentum vector is represented in the matrix form as:

(14) $$h_{g}=h_{0}\;\left[\begin{array}{c} -\sin\alpha \\ \cos\alpha \cos\beta \\ -\cos\alpha \sin\beta \end{array}\right]$$

where *h*_0_ is the angular momentum magnitude of the individual MSDGCMG, *α* and *β* are the inner and outer gimbal angle, respectively.

Therefore, the MSDGCMG output torque vector *ḣ_g_* is derived as:

(15) $${\dot{h}}_{g}=h_{0}\cdot C\cdot \dot{\delta }$$

where *δ̇* = [*α̇ β̇*]*^T^* is the gimbal angle rate, *C* is the Jacobian matrix:

(16) $$C=\left[\begin{array}{cc} -\cos\alpha & 0\\ -\sin\alpha \cos\beta & -\cos\alpha \sin\beta \\ \sin\alpha \sin\beta & -\cos\alpha \cos\beta \end{array}\right]$$

It is clearly seen from the above equation that, the Jacobian matrix is a nonlinear function of the gimbal angles *α* and *β*.

### 4.3. MSDGCMG Steering Law

For the attitude control of the single axis air bearing table, particular attention should be paid to the MSDGCMG output torque along the pitch axis. Therefore *A* is written by extracting the second row of Jacobian matrix *C*:

(17) $$A=\left[\begin{array}{cc} -\sin\alpha \cos\beta & -\cos\alpha \sin\beta \end{array}\right]$$

The command gimbal rate *δ̇*, referred to as the pseudoinverse steering law, is then obtained as:

(18) $$\dot{\delta }=-A^{+}u/h_{0}\;\text{where}\;A^{+}=A^{T}{\left(AA^{T}\right)}^{-1}$$

where *u* denotes the command control torque derived by the attitude control law in the preceding section.

For the reason that the high-rate magnetically suspended rotor of the MSDGCMG is actually active-control elastic supporting with certain stiffness and damping, the gimbal movement will disturb the magnetically suspended rotor. For simplicity, the phenomena caused by the gimbal movement are called the moving-gimbal effects. On the other hand, the response bandwidth of the CMG gimbal is constrained by the output torque ability of gimbal motor, thus the gimbal servo systems are unable to respond to the large gimbal angle acceleration. Consequently, the limit of the gimbal angle velocity and the acceleration should be given full consideration, to make sure that the high-speed rotor would work steadily around the operating point.

Furthermore, the singularity problem is inherent in the CMG. When the MSDGCMG is trapped in the singular state, the gimbal of the MSDGCMG will vibrate seriously so as to influence the stability of the MSDGCMG. Therefore, the maximum gimbal angle acceleration *δ̈*_max_ is determined on the basis of the singularity measurement:

(19) $${\ddot{\delta }}_{\max}=\{\begin{array}{lll} {\ddot{\delta }}_{\max1} & if & 0<D<d\\ {\ddot{\delta }}_{\max2} & if & D\geq d \end{array}$$

where *D* = det(*AA^T^*) is the singularity measurement, *d* is the piecewise point.

In the experiment, the maximum limit of the gimbal angle acceleration is firstly taken into consideration. The inner and outer gimbal angle acceleration *δ̈_k_* at moment *k* is calculated as:

(20) $${\ddot{\delta }}_{k}=({\dot{\delta }}_{k}-{\dot{\delta }}_{k-1})/T_{s}$$

where *δ̇_k_* and *δ̇_k_*_−1_ are the command gimbal angle rates at moment *k* and *k* − 1, respectively. *T*_s_ denotes the control cycle. Then the gimbal angle acceleration is constrained as follows:

(21) $${\ddot{\tilde{\delta }}}_{k}=\{\begin{array}{cc} {\ddot{\delta }}_{k} & \begin{array}{cc} if & {\left\|{\ddot{\delta }}_{k}\right\|}_{\infty }<{\ddot{\delta }}_{\max} \end{array}\\ {\ddot{\delta }}_{k}\frac{{\ddot{\delta }}_{\max}}{{\left\|{\ddot{\delta }}_{k}\right\|}_{\infty }} & \begin{array}{cc} if & {\left\|{\ddot{\delta }}_{k}\right\|}_{\infty }\geq {\ddot{\delta }}_{\max} \end{array} \end{array}$$

where ‖•‖_∞_ is the infinite norm of the vector, and then the command gimbal rate at the moment *k* is modified as:

(22) $${\dot{\delta }}_{k}={\dot{\delta }}_{k-1}+{\ddot{\tilde{\delta }}}_{k}\cdot T_{s}$$

Finally, the gimbal angle velocity is calculated as follows:

(23) $${\dot{\tilde{\delta }}}_{k}=\{\begin{array}{cc} {\dot{\delta }}_{k} & \begin{array}{cc} if & {\left\|{\dot{\delta }}_{k}\right\|}_{\infty }<{\dot{\delta }}_{\max} \end{array}\\ {\dot{\delta }}_{k}\frac{{\dot{\delta }}_{\max}}{{\left\|{\dot{\delta }}_{k}\right\|}_{\infty }} & \begin{array}{cc} if & {\left\|{\dot{\delta }}_{k}\right\|}_{\infty }\geq {\dot{\delta }}_{\max} \end{array} \end{array}$$

where *δ̇*_max_ denotes the allowed maximum gimbal angle rate. Then the command gimbal angle rate 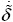
*_k_* is derived from Equation (23) taking account of the limit of the gimbal angle rate and acceleration.

Note that the maximum limit of the gimbal angle rate and the acceleration defined as above has the same direction as that of themselves before maximum limit. That is, the MSDGCMG output torque is kept the same direction as the command control torque, but the magnitude of the actual torque is reduced.

## 5. Design of the Semi-Physical Simulation Platform for MSDGCMG Performance Testing

The design of semi-physical simulation platform for MSDGCMG performance testing is introduced in this section. The single axis air bearing table is used to simulate the pitch axis of the satellite. The MSDGCMG is mounted on the air bearing table and is used as an attitude actuator to realize rapid maneuvers and quick stability of the air bearing table.

**Figure 2.**
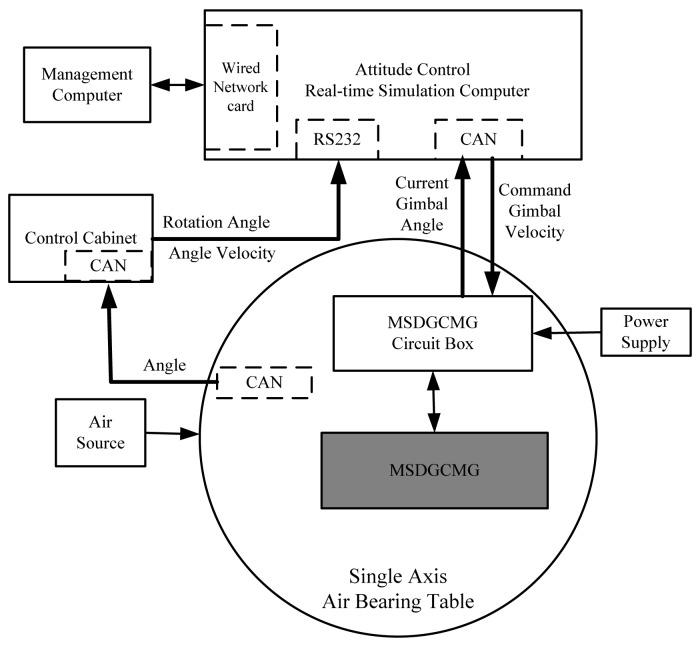
Hardware Layout Frame of Semi-Physical Simulation System.

The hardware used in the semi-physical simulation platform is mainly composed of the MSDGCMG, the corresponding circuit box, single axis air bearing table, control cabinet of the air bearing table, attitude control real-time simulation computer, simulation management computer, power module, and so on. The hardware layout frame of the semi-physical simulation system is depicted in Figure 2.

The signal flow of the hardware is described as follows: the rotation angle of the air bearing table is measured by the photo-electric encoder fixed to the single axis air bearing table, and then is delivered to the control cabinet through CAN bus. The control cabinet collects the rotation angle information, derives the angle velocity through differential filtering of the rotation angle, and then delivers the rotation angle and angle velocity to the real-time simulation computer through the RS232 serial port. According to the error-angle between the current rotation angle and the command angle, the command torque is generated through the parameter segment control algorithm. In light of the command torque and the current MSDGCMG gimbal angle, the command gimbal rate is computed and is sent to the MSDGCMG circuit box through the CAN bus. The circuit box receives the command and then controls the gimbal servo system to rotate the gimbals. Because of the change of the direction of CMG angle momentum, the desired control torque of the MSDGCMG is finally exerted on the air bearing table.

In this experiment, the single axis air bearing table is the supporting platform of the attitude control semi-physical simulation system and the main parameters are described as:

1. Loading capacity: >200 Kg.
2. Friction torque: <5 × 10^−4^ Nm.
3. Rotation angle range: ±360°.
4. Rotation angle measurement accuracy: ±10^−3^°.
5. Rotation angle minimum resolution: 2.5 × 10^−4^°.

The attitude control real-time simulation system is a hardware-in-loop semi-physical simulation, which supports the Matlab/Simulink graphical module design, seamless connection with C coder, and automatic production of code. The hardware of this system is mainly composed of the simulation management computer (host computer), real-time simulation computer (target computer) and signal processing box. The main function of host computer here is to manage the real-time target computer. The host computer is based on the Windows platform, and Matlab/Simulink is applied for building the simulation model. The target computer adopts the Vxworks real-time operating system to guarantee the reliability of the semi-physical simulation. The communication between the host computer and the target computer is by means of Ethernet networks. What's more, the major role of the signal process box is to provide the signal processing and connection to the I/O interface, and to ensure the data communication between the semi-physical simulation platform and the hardware equipment.

## 6. Experimental Results and Analysis

**Figure 3.**
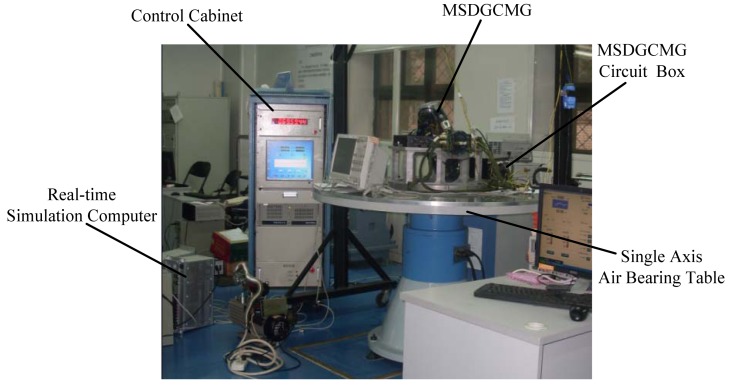
Experiment Scene of Semi-Physical Simulation.

Having designed the attitude control law, the MSDGCMG steering law as well as the semi-physical simulation platform, experiments are conducted in this section to validate the performance of the MSDGCMG. The following mainly includes the measurement of the moment of inertia, the measurement of the disturbance torques, experimental results and analysis. The experimental scenario is given in Figure 3.

### 6.1. Measurement of the Moment of Inertia

The measurement of the air bearing table moment of inertia is important in the initial stage of the semi-physical simulation. Here the twisting vibration approach [18] is applied for measuring the moment of inertia. This method has the advantages of simple design, easy operation, as well as not needing any knowledge of the air bearing table mass. Only the spring resonance device is needed in our experiment.

During the measurement of the moment of inertia, it should be ensured that the vibration amplitude of the air bearing table is small to satisfy the condition of micro-amplitude. However, the equivalent torsional stiffness is difficult to measure, and the change of torsional stiffness resulting from the reinstallation brings about large error in the moment of inertia. In order to obtain the moment of inertia *J* precisely, two standard cylinder balance masses are symmetrically placed on the air bearing table, and then the air bearing table moment of inertia is given as:

(24) $$J=\frac{f_{2}^{2}}{f_{1}^{2}-f_{2}^{2}}\cdot \Delta J$$

where *f*_1_ denotes the system vibration frequency without the balance mass, *f*_2_ denotes the system vibration frequency when the air bearing table is installed with the balance masses, Δ*J* is the increased moment of inertia:

(25) $$\Delta J=2m_{0}L^{2}+m_{0}r^{2}$$

where *m*_0_ and *r* are the mass and the radius of the balance mass, respectively. *L* represents the distance from the rotating axis of the air bearing table to the centre of the balance mass.

Here the vibration period is calculated by counting the zero-crossing number of the air bearing table angle velocity within a specified time. Multiple measurements are carried out, and an average measurement value is used from ten attempts. The experimental data is shown in Table 1:

### 6.2. Measurement of the Disturbance Torques

In the experiment, the air bearing table is subjected to some external disturbance torques, such as gravity torque resulted from incomplete balancing, friction torque produced from air bearing, the resistance moment caused by communication cable and power supply cable, and so on. Taking account of the influence of the disturbance torques on the control accuracy and attitude stability, the disturbance torque *T_d_* is calculated and the equation is given as follows [19]:

(26) $$T_{d}=J\frac{\Delta \omega }{\Delta t}$$

where Δ*ω* denotes the change of angle velocity during time Δ*t*.

Through multiple measurements, the average value of the disturbance torque is measured as 1.09 × 10^−4^ Nm.

### 6.3. Experimental Results and Analysis

In the experiment, the parameter segment attitude control law developed in this paper is now integrated to the MSDGCMG steering law. The parameters shown in the preceding sections are properly selected as in Table 2.

**Figure 4.**
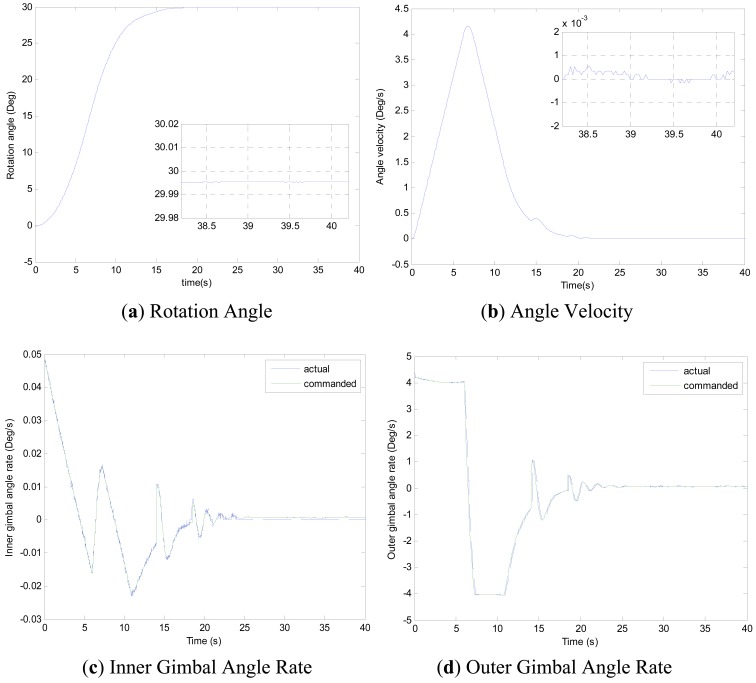

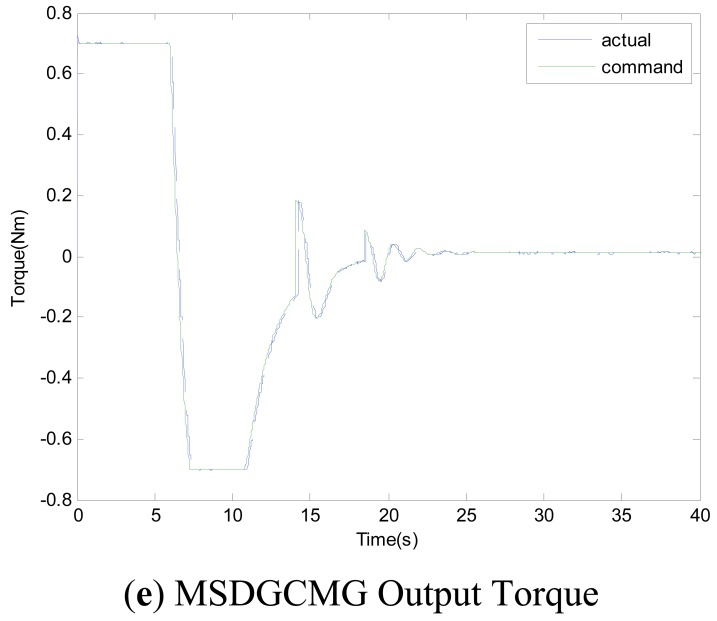
MSDGCMG Rotor Speed 6,000 rpm (Case 1).

**Figure 5.**
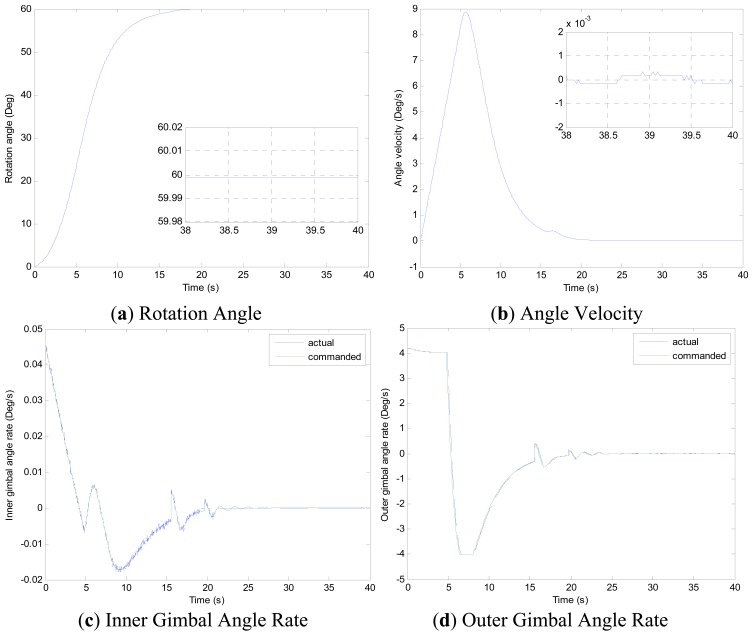

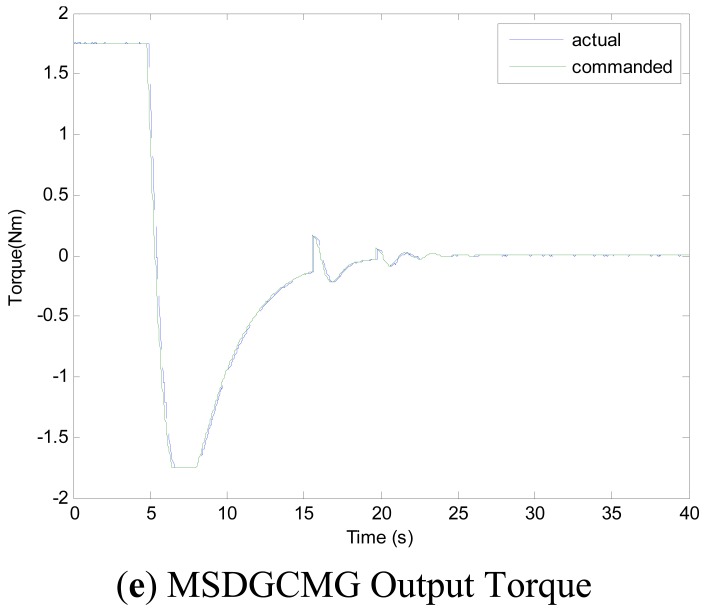
MSDGCMG Rotor Speed 15,000 rpm (Case 2).

**Figure 6.**
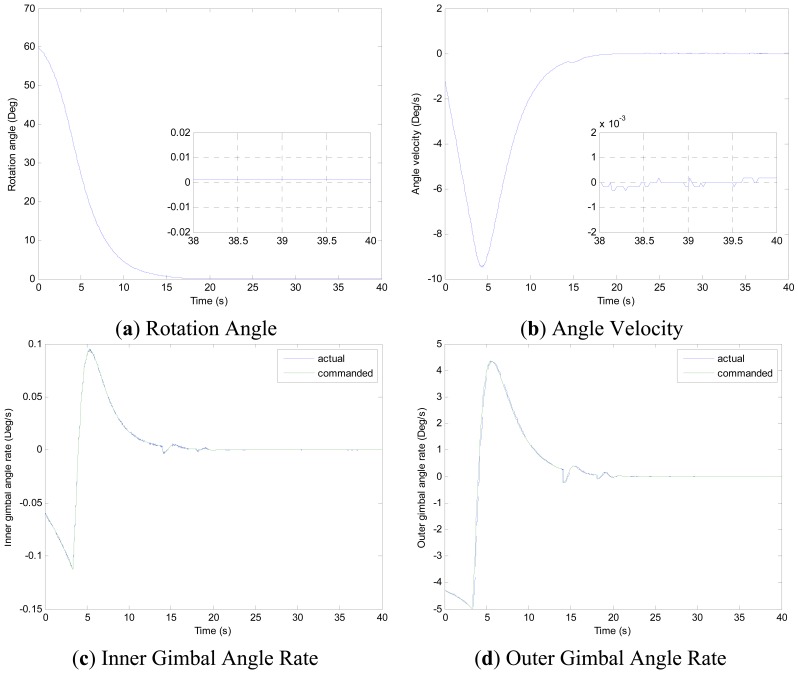

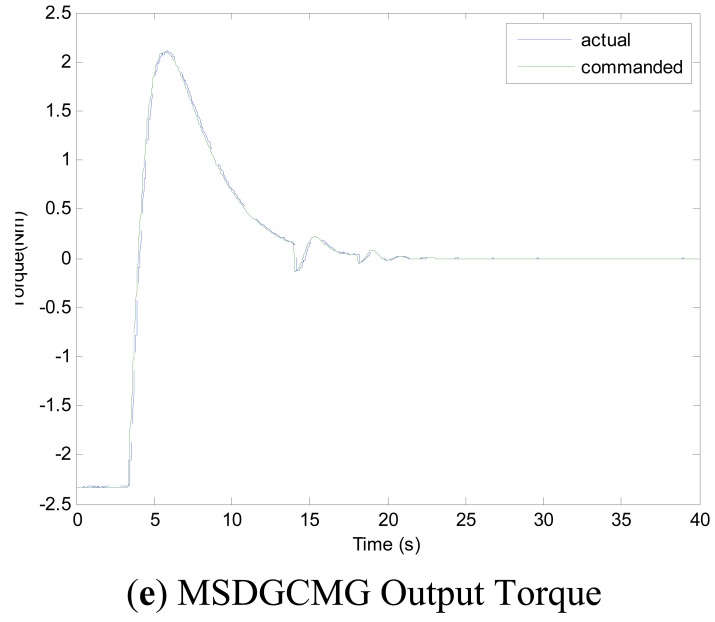
MSDGCMG Rotor Speed 20,000 rpm (Case 3).

Experiments of the following three cases are carried out with different MSDGCMG rotor speed and different command rotation angles of the air bearing table. Case 1, as shown in Figure 4, is to realize a 30° attitude maneuver with 6,000 rpm rotor speed of the MSDGCMG. In this case, the initial air bearing table angle is 0°, and the command angle is set at 30°. Case 2, as shown in Figure 5, is to realize 60° attitude maneuver with 15,000 rpm rotor speed. In this case, the initial angle is 0°, and the command is 60°. Case 3, as shown in Figure 6, is to realize 60° attitude maneuver with 20,000 rpm rotor speed. In this case, the initial angle is 60°, and the command is 0°.

From the experimental results of the three cases, it is indicated that the larger the rotor speed (angular momentum of the MSDGCMG) is, the shorter the time required to realize the same angle maneuver is, meaning that the greater the maneuver ability is. It's easily known from the above figures that, the inner and outer gimbal angle rates follow the command perfectly, and that MSDGCMG output torque almost agrees with the command torque under the moving-base condition. Moreover, the attitude stability with the MSDGCMG as an actuator is superior to 10^−3^°/s, and there exists no transient overshoot during the process of the rapid maneuver and quick stability.

It is clearly seen from the Table 3 that, the MSDGCMG angle momentum determines the maneuver velocity, and that the maximum average maneuver ability is up to 3.57°/s. For the reason of the coupling between the air bearing table, MSDGCMG gimbal and high-rate rotor, the quicker the angle velocity of the air bearing table is, the more serious the influence on the high-rate magnetically suspended rotor becomes. In the experiment, for the reason of the high-precision magnetic bearing control, the MSDGCMG is able to overcome the influence that moving-base effects bring about, even when the maximum angle velocity reaches to 9.49°/s and the speed of high-rate rotor is 20,000 rpm.

In the future, advanced control algorithms [20–22] can be employed to further improve the control performance.

## 7. Conclusions

In order to fully validate the performance of the MSDGCMG developed by Beihang University, experiments under the moving-base condition are carried out, with different MSDGCMG rotor speeds and different command rotation angles. Here a parameter segment control law is used for the air bearing table rapid maneuver and quick stability subject to the output torque saturation limit. Meanwhile, in view of the moving-gimbal effects on the stability of the magnetic bearing and the gimbal response bandwidth limit, a special steering law is presented subjected to the limit of MSDGCMG gimbal angle rate and acceleration. Experimental results illustrate that the attitude stability of attitude control system is superior to 10^−3^°/s by using the MSDGCMG and that the MSDGCMG output torque follows the command torque well. Moreover, it is drawn from the experiments that the larger the angle momentum is, the greater the maneuver capacity is. However, when the MSDGCMG gimbal quickly revolves and the satellite rapidly maneuvers, the moving-gimbal effects become strikingly serious. Therefore, in the future research, not only the limit of gimbal angle rate and acceleration should be taken consideration, but also particular attention should be paid to the limit of attitude velocity when an attitude control law is designed. These are the difference between the application of a mechanically suspended DGCMG and that of the MSDGCMG. The MSDGCMG performance testing in this paper, carried out under moving-base conditions, will offer a technical base for the future research and application of MSDGCMGs.

## Figures and Tables

**Table 1.** Experimental Data of the Moment of Inertia.

**Balance Mass**	**0**	**Mass: 9.79 kg; Radius: 0.1 m; Distance from Center: 0.65 m**				**Average**
Vibration Amplitude (°)	1.03	1.100	1.040	1.090	1.120	——
Frequency (Hz)	0.32	0.297	0.297	0.297	0.297	——
Moment of Inertia (kgm^2^)	——	53.976	54.026	54.113	54.088	54.051

**Table 2.** Parameters and Values of Experiment.

**Parameters**		**Value**
Parameter Segment Control Law	*k*	*k*_1_ = 25, *k*_2_ = 50, *k*_3_ = 100
	*a*	*a*_1_ = 1, *a*_2_ = 2, *a*_3_ = 3
	*ε*	0.5
	*g*	0.001
	Piecewise points of error-angle *θ_e_*	*θ_e_*_1_ = 0.1°, *θ_e_*_2_ = 0.01°
	Saturation limit of control torque *U*_max_	Rotor speed 6,000 rpm: 0.70 Nm
		Rotor speed 15,000 rpm: 1.75 Nm
		Rotor speed 20,000 rpm: 2.30 Nm
	Control cycle *T*_5_	0.05 s
MSDGCMG Steering Law	Maximum gimbal angle rate *δ̇*_max_	6°/s
	Maximum gimbal angle acceleration *δ̈*_max_	*δ̈*_max1_ = 5.73°/s^2^, *δ̈*_max2_ = 30°/s^2^
	Piecewise points of singularity measurement *d*	0.05

**Table 3.** Experimental Results of Three Cases.

**Evaluation Items**	**Attitude Stability (°/s)**	**Pointing Accuracy (°)**	**Settling Time (s)**	**Average Maneuver Ability (°/s)**	**Maximum Angle Velocity (°/s)**
Case 1	0.51 × 10^−3^	0.0035	18.35	1.63	4.16
Case 2	0.34 × 10^−3^	0.0011	18.85	3.18	8.88
Case 3	0.18 × 10^−3^	0.0012	16.80	3.57	9.49
